# Pathways into single motherhood, re-partnering, and trajectories of antidepressant medication purchases

**DOI:** 10.1007/s00127-022-02371-2

**Published:** 2022-10-13

**Authors:** Mine Kühn, Niina Metsä-Simola, Pekka Martikainen

**Affiliations:** 1 Max Planck Institute for Demographic Research, Konrad-Zuse-Straße, 18057 Rostock, Germany; 2 Population Research Unit, University of Helsinki, P.O. Box 18, N00014 Helsinki, Finland; 3 Department of Public Health Sciences, Stockholm University, Stockholm, Sweden

**Keywords:** Single motherhood, Pathways into single motherhood, Mental health, Antidepressant purchases, Finish registry data

## Abstract

Single motherhood is known to be distressing, and to be associated with poor mental health. However, less is known about the pathways into and out of single motherhood, or about the mental health trajectories of single mothers. We used total population registry data on Finnish women who experienced the life events of separation (616,762), widowhood (43,355), or child birth (515,756) during the 1995–2018 period while between the ages of 15–64. Single mothers were compared with women who experienced the same life event, but without becoming a single mother. The results for women who separated showed that among single mothers, there was a substantial increase in antidepressant use at the time of separation, and only a moderate decline after separation. Among women who experienced widowhood, those who had underage children initially had lower antidepressant use than women without children, but this gap narrowed in the post-widowhood period. In addition, single women experienced more unfavorable mental health trajectories than partnered women around the time they gave birth. Re-partnering was associated with more favorable mental health among all groups of single mothers. Given the growing prevalence of single-parent households, our results underscore the need for context-specific interventions to support single mothers’ mental health.

**Supplementary Information:**

The online version contains supplementary material available at 10.1007/s00127-022-02371-2.

## Introduction

A large body of studies has demonstrated that single mothers have worse psychological well-being [1], a higher prevalence of depressive symptoms [2–6], more psychological distress [7, 8], and more anxiety disorders [9, 10] than partnered mothers. While these findings have been consistent, most previous studies on this topic assessed single mothers’ health at a single point in time, while overlooking the possibility that pathways into single motherhood and later partnership status could prove to be important factors in determining the mental health trajectories of single mothers.

This paper draws on theoretical insights from scholarship on social stress and the life course to examine the effects on women’s mental health of becoming a single mother and of being a single mother. While most previous studies have focused on cross-sectional comparisons of single mothers with partnered mothers, we investigate three pathways into single parenthood: separation, widowhood, and giving birth while single, and compare the prevalence of antidepressant use among single mothers before and after they became a single mother with that of women who experienced the same life event, but without becoming a single mother. This approach enables us to compare the mental health trajectories associated with different pathways into single motherhood, and to disentangle the effects of these life events on women’s mental health from the effects of being a single mother. To evaluate the impact of re-partnering on the mental health of women, we also compare women who stayed single and women who re-partnered.

We use high-quality longitudinal registry data from Finland, which ensures that we have high sample numbers for each group of women. As single mother households emerged relatively early in Finland, and the country continues to have a high prevalence of single mother households [11], Finland can be seen as a forerunner in trends that arrived later in other countries, or contrasted with countries where single motherhood remains rare. Nordic countries, including Finland, have a reputation for being welfare states with generous family policies and relatively high employment rates among single mothers. However, even in these countries, increasing educational gradients and employment gaps between single mothers and partnered mothers have emerged in recent decades [12]. The disadvantaged socioeconomic status of single mothers in Finland might be reflected in their mental health as well.

When examining mental health from the life course perspective, it is essential to look at individual lives as a series of interconnected processes and transitions, and to take the effects of various life events into account, as they may have important implications for mental health. Although the mental health effects of separation, widowhood, and childbirth have been previously documented, little is known about the mental health trajectories of women who enter single motherhood through these life events. One of the pathways into single motherhood is through separation. Studies that have looked at the consequences of separation and divorce have consistently found that these events have negative effects on the ex-partners’ stress levels and health [13–17]. There is empirical evidence that the health disadvantage of single mothers is partly attributable to the chronic economic strains and other social stressors associated with sole parenting responsibilities [3, 18]. Furthermore, as these stressors have been found to persist over time, it appears that their effects are not transitory [19].

Based on these theoretical and empirical considerations, we expect to find that the mental health of all women who experienced separation declined. Furthermore, because single mothers had ongoing exposure to post-separation stress, we expect to observe a steeper decline and a slower recovery in mental health after separation for women who entered single motherhood than for women who did not have underage children (Hypothesis 1a).

In some cases, the children of a separated mother are living with their father rather than with her in the post-separation period. However, while the number of single-father households has been increasing and the prevalence of the gendered division of labor has been decreasing, the vast majority of single-parent households are still headed by women. Thus, cultural norms about a woman’s role as a mother may result in additional stressors for mothers who do not live with their children, even in highly equitable countries like Finland. Furthermore, as poor mental health could be one reason why a mother’s children do not live with her, there may be negative selection into this group. Therefore, we expect to find that mothers who were not living with their children following their separation had worse mental health than separated women without children and single mothers throughout the observation window (Hypothesis 1b).

While much less common than separation or divorce, another pathway into single motherhood is through widowhood. There is consistent evidence that widowhood is one of the most stressful events that individuals can experience over the life course [20], albeit with greater short-term than long-term effects on health [21, 22]. The death of a partner has been shown to be a disruptive life stressor [23, 24] that often requires a restructuring of the widow’s day-to-day life [24, 25]. As the timing principle of the life course perspective underscores the importance of considering when events and transitions occur within an individual’s personal biography, a woman’s age and whether she has underage children when she becomes a widow are key aspects of the timing of this life course event that may have an impact on the woman’s mental health. In addition, experiencing a traumatic loss and becoming a single mother might be associated with additional stressors. We assume that all women who are widowed experience a decline in mental health. However, as a single mother is likely to face greater changes in her daily life than a widow with no underage children, we expect to find that widowed single mothers had especially poor mental health in the post-widowhood period (Hypothesis 2a).

Another increasingly important pathway into single motherhood is giving birth as a single woman. This transition occurs in a different context than separation and widowhood, as some of these women may be single mothers by choice. The birth of a child represents a crucial moment in a woman’s life course [26], and many studies have found that depressive disorders are common during pregnancy and in the postpartum period [27, 28]. Little is known about whether mothers who give birth while single are at increased risk of experiencing adverse mental health outcomes [29, 30]. However, research has indicated that spousal support can have a protective effect and reduce stress in the transition to motherhood [31]. Based on the assumption that women who entered single motherhood through giving birth have less support than partnered mothers, we expect to find that these women had poorer mental health than partnered women during pregnancy, during the year of the birth, and in the post-birth years (Hypothesis 3a).

In addition to differing in their pathways into single parenthood, single mothers also differ with regard to their partnership status later in life. As the theory and research on stress and the life course perspective have highlighted, an individual’s life should be seen as consisting of dynamic relationships and social processes that unfold over time [32, 33]. To understand the health implications of experiencing single motherhood, it is important to take re-partnering into account, as knowing whether and when a woman re-partners provides information about how long she was a single mother, and thus about the length of her exposure to stressors that could negatively affect her mental health [34–36].

The limited evidence on the health effects of re-partnering indicates that both previously divorced and widowed women who re-partner are healthier than those who remain single [37]. While this finding is partially attributable to selection effects—i.e., healthier widows and separated women are more likely to re-partner than their less healthy counterparts [38, 39]—being in a relationship also provides many economic, social, and psychological resources that enhance well-being [40]. Re-partnering may reduce many of the stressors associated with being a single mother. Becoming a single mother is often accompanied by a deterioration in a woman’s economic situation [41], and an increase in her financial resources through re-partnering may offset the stress caused by economic insecurity, which could have beneficial effects on her psychological well-being [18]. The new partner may also be an important source of help in dealing with the role overload that many single mothers suffer from [42]. By sharing the day-to-day demands of childcare, a single mother’s new partner may help to reduce her parenting stress, which could, in turn, improve her mental health [43]. It has been also shown that experiencing emotional warmth and sexual intimacy is associated with better mental health [44, 45]. Thus, we expect to observe more favorable mental health outcomes for separated women (Hypothesis 1c), for widowed women (Hypothesis 2b), and for women who gave birth while single (Hypothesis 3b) who re-partnered than for women who remained single.

## Methods

We used register-based data on all Finnish women who were aged 0–55 years during the 2000–2015 period. The data for this period are the most recent that are currently available. Thus, the evidence we present here is as up-to-date as possible. We drew from these data information on women who were aged 15–64 from 1995 to 2018. The Finnish data provide information on all of the children who were born to these women, on the women’s dates of entry into and exit out of marital and non-marital cohabitation, and on their annual socio-demographic characteristics. These data are linked to information on medication purchases and reimbursements by the Social Insurance Institution. The data linkage was done by Statistics Finland using personal identification codes (the Ethics Committee of Statistics Finland’s permission TK-53-1121-18).

The mental health of the women in our sample was measured based on their antidepressant purchases (ATC codes N06A). In Finland, antidepressants are prescribed by general practitioners, and all residents of the country are entitled to reimbursement for medication expenses [46–48]. It has been shown that in Finland, the probability of antidepressant use is similar irrespective of individuals’ education, income, employment status, and living arrangements [49]. This suggests that any changes in antidepressant use accurately reflect underlying changes in depression, without causing bias. Furthermore, studies have found that antidepressant use predicts the likelihood of having other negative outcomes, including retiring due to disability and mortality, which underscores the relevance of studying changes in antidepressant use [50, 51]. The prescription register includes information on the dates of medication purchases, and on the types of medications purchased.

An episode of single motherhood began in the year (a) when the partner moved out of the household and the mother was living alone with her underage biological child/ren; (b) when the partner died and the mother was living alone with her underage biological child/ren; or (c) when a woman gave birth and was not living with a partner. For women who experienced multiple events,^1^ we included the first event of each type. For all women, we also observed whether they re-partnered within five years of the event using information on whether and when they entered into marital or non-marital cohabitation.

To identify the effects of the different pathways women took into single motherhood, and the effects of women’s subsequent re-partnering behavior on their mental health trajectories, we followed them three years before and five years after they entered single motherhood. Examining the period three years before the event occurred provided us with sufficient information on potential selection processes, as well as with information on the potential anticipation of conflicts (separation) and on stressors due to spousal illness (widowhood). By studying women’s mental health trajectories in the five years immediately after the event we were able to focus on relatively short-term changes, and thus to avoid the endogeneity issues associated with longer observation periods.

The episodes were right-censored at the end of the observation period on December 31, 2017, or upon emigration, entry into institutional care, or death. As the women in the samples were relatively young, and only a small percentage were migrants, censoring was likely to be independent of the event, and was unlikely to lead to a substantial underestimation of antidepressant use. For each year, we identified whether any purchases of antidepressants had been made.

The annual probability of making antidepressant purchases was modeled using logistic regression with an outcome of 1 if women had been prescribed antidepressant use during a 12-month observation window, and of 0 otherwise. Using generalized estimation equations with an unstructured correlation matrix, logistic regression models controlled for correlations within individuals [52]. The results are presented as trajectories of the predicted annual antidepressant prevalence.

All models were adjusted for age, calendar year, education (tertiary, secondary, basic, or unknown; based on the highest educational qualification), individual disposable income (annual quintiles in the sample of all women), employment (yes or no), unemployment (yes or no), country of birth (Finland vs. other), and municipality group (urban, semi-urban, rural). Supplementary Table S1 provides descriptive statistics by the life events the women experienced and their family status at the time of the events.

## Results

Our study population included 616,762 separated women, 43,355 widowed women, and 515,756 women who gave birth during the follow-up (Table 1). Of the separated women, 38% had biological children, while 62% had no underage biological children. Of the separated women with biological children, the majority entered single motherhood (89.7%), while 10.3% were not living with their child/ren following their separation. Of these single mothers, 54.3% remained single for the next five years, while 45.7% re-partnered within the same observation window.

**Table 1:** Life events, family status, and pathways into and out of single motherhood, Finland, 1998–2015

Life events (t0)	Family status at the time of the event	Status during the five years after the event	*N*	%	AD prevalence t0
Separation					
	Has biological child(ren)				
		Lives with children, single	108,155	17.23	14.9
		Lives with children, re-partnered	91,201	14.43	12.5
		Does not live with children, single	18.58	2.94	20.7
		Does not live with children, re-partnered	22,798	3.61	16.8
	No underage biological child	Single	177,434	27.44	15.3
		Re-partnered	217,155	34.35	9.8
N			616,762	100	
Widowhood					
	Has biological child(ren)	Single	7117	16.42	17.2
		Re-partnered	2018	4.65	18.9
	No underage biological child	Single	30,160	69.57	18.7
		Re-partnered	4060	9.36	22.5
N			43,355	100	
Giving birth					
	Single	Single	24,243	4.7	6.9
		Partnered	25,103	4.9	4.9
	Partnered	Partnered	409,030	79.3	2.7
		Separated	57,380	11.1	5.6
N			515,756	100	

Of the women who experienced widowhood, 78.9% had no underage biological children and 21.1% entered single motherhood. Most of these single mothers stayed single (88.1%), while 11.9% re-partnered.

The vast majority of the women who gave birth were partnered (90.4%), while only 9.4% gave birth without having a partner in the same household. Of these single mothers, 49.1% stayed single and 50.9% partnered during the next five years. Of those who gave birth while being partnered, 12.3% separated and entered single motherhood.

The women also varied with regard to their socio-demographic profiles. Separated mothers who were not living with their children had lower incomes than separated mothers who were living with their children, but higher incomes than separated women without children. Separated mothers who were not living with their children and stayed single were the most likely to be non-employed and to have an immigration background, whereas separated women who had no children and who re-partnered were the most likely to be employed (80%), and were clearly younger than all other groups of separated women. Widowed women who were single mothers were younger and had higher levels of education, income, and employment status than widowed women without underage children. Among the women who gave birth, partnered women who stayed partnered had better socio-demographic profiles than their single counterparts (see Supplementary Table S1).

### Mental health around the time of separation

**Fig. 1:**
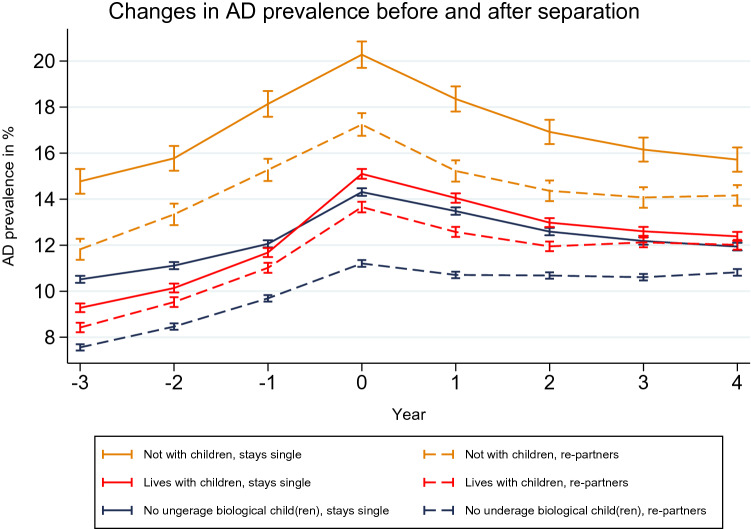
Trajectories of predicted annual antidepressant (AD) prevalence of women separating at t_0_, Finland, 1998–2015. *Model adjusted for age, calendar year, education, individual disposable income, country of birth, and municipality group

Figure 1 plots the results of the logistic regression model predicting the annual antidepressant prevalence. On the x-axis, the years before, during, and after separation are plotted. The y-axis shows the predicted antidepressant prevalence for the separated women in our sample. We present the results of the full regression models (the β coefficients) in the supplement of this paper (Table S3–S5).

Antidepressant use clearly started to increase during the 3 years before separation, and reached a peak at the time of separation. After separation, the prevalence decreased continuously, and remained relatively stable after 2 years of separation. The prevalence of antidepressant use was higher in the post-separation period than in the pre-separation period. While this pattern was observed for all groups of separated women, the levels of antidepressant use differed across the groups.

Among separated women, those who entered single motherhood and stayed single after separation experienced the steepest increase in antidepressant use in the pre-separation period, with their use reaching a peak of 15.1% (95% CI 14.89–15.31) at the time of separation. While the level of antidepressant use three and two years prior to separation was significantly lower among these women than it was among women who had no biological children, the differences in their antidepressant use levels narrowed and became non-significant in the year before separation, which suggests that women with children suffered more from stressors due to conflicts that occurred immediately before the separation. Thereafter, single mothers’ antidepressant use increased steeply, and became significantly higher than that of women who had no biological children at the time of separation and in the first two years following separation. These findings reflect the mental health disadvantage for single mothers that was predicted in Hypothesis 1a.

The highest levels of antidepressant use were found for mothers who were not living with their children following their separation. Their antidepressant prevalence differed significantly from that of other separated women before, during and, after the separation. These findings are in line with the expectations we formulated in Hypothesis 1b.

In Hypothesis 1c, we predicted that the women who re-partnered would have better mental health than their counterparts who remained single. Our results show that women who had no biological children under age 18 at the time of separation and who re-partnered had the lowest levels of antidepressant use, with a peak prevalence of 11.2% (95% CI 11.06–11.35). Single mothers who re-partnered had significantly lower levels of antidepressant use than single mothers who stayed single. This gap became more pronounced at the time of separation and in the years immediately after the separation. There were also significant differences in the antidepressant trajectories of mothers whose children were not living in the same household. Re-partnering was associated with lower levels of antidepressant use (peak prevalence of 17.2% (95% CI 16.75–17.73)) than staying single in the next five years (peak prevalence of 20.3% (95% CI 19.71–20.85)). Thus, our findings indicate that there were mental health differences between women who re-partnered and women who remained single, in line with Hypothesis 1c.

### Mental health around the time of widowhood

**Fig. 2:**
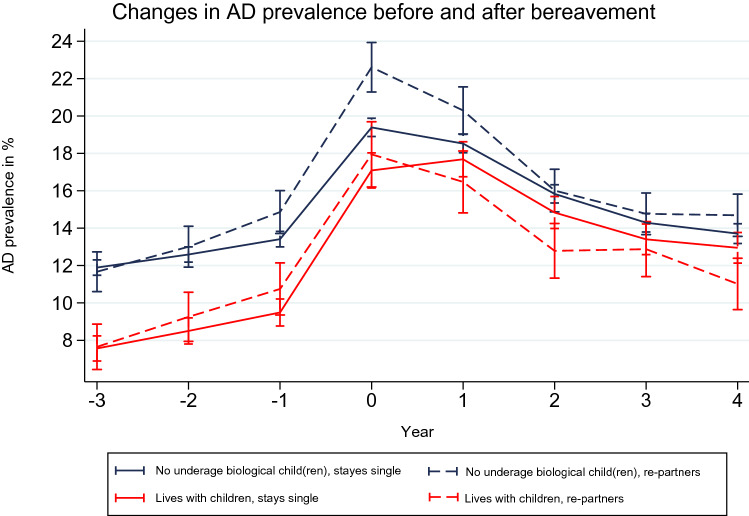
Trajectories of predicted annual antidepressant (AD) prevalence of women becoming widowed at t_0_, Finland, 1998–2015.*Model is adjusted for age, calendar year, education, individual disposable income, country of birth and municipality group

Figure 2 shows the antidepressant trajectories for women who experienced widowhood with and without having children under age 18. For all widowed women, the prevalence of antidepressant use increased monotonically from a low base in the three years before the death of their spouse. Their levels of antidepressant use increased sharply in the year before the death, and reached a peak in the year when their spouse died. For all widowed women, the prevalence decreased constantly after the first year of widowhood.

Widowed single mothers had significantly lower levels of antidepressant use than widows without underage biological children in the pre-widowhood period. However, these differences between widowed single mothers and widowed women with no underage biological children narrowed after the spousal death, and became non-significant. The less steep decline in antidepressant use after the spousal death observed among single mothers contributed to a narrowing of the gap in antidepressant use for all women in the post-widowhood period. From these findings, we conclude that our expectations formulated in Hypothesis 2a could be partially validated by our data, as the increase in antidepressant use was steeper for single mothers before they entered widowhood, and was more persistent in the post-widowhood period than it was for widowed women with no underage biological children. However, the levels of antidepressant use among widowed single mothers were not higher than those among widows without underage biological children, as was suggested in Hypothesis 2a.

The mental health of widowed single mothers who re-partnered did not differ significantly from that of widowed single mothers who stayed single. While the finding was non-significant, re-partnered single mothers had an even higher prevalence of antidepressant use in the years before and in the year when they became a widow than widowed single mothers who stayed single. This pattern changed in the first post-widowhood year. While the antidepressant use of single mothers who did not re-partner increased, that of single mothers who re-partnered declined. In the following years, the levels in antidepressant use were higher for single mothers who remained single than for those who re-partnered, but the differences were non-significant.

Widowed women without children who re-partnered had, at 22.6% (95% CI 21.28–23.93), a higher prevalence of antidepressant use at the time of widowhood than all other widowed women. However, as their antidepressant use declined steeply between one and two years after the death of their spouse, the difference between their use and that of other widowed women became non-significant two years later. Taken together, these findings do not show a clear pattern of widows who re-partnered having a mental health advantage compared to those who stayed single throughout the observation window. However, we observed a steeper decline in antidepressant use among both re-partnered single mothers and re-partnered women without underage biological children than among widows who stayed single in the period following the death of their spouse. These findings point to the mental health benefits of re-partnering, and thus support Hypothesis 2b.

### Mental health around the time of giving birth

**Fig. 3:**
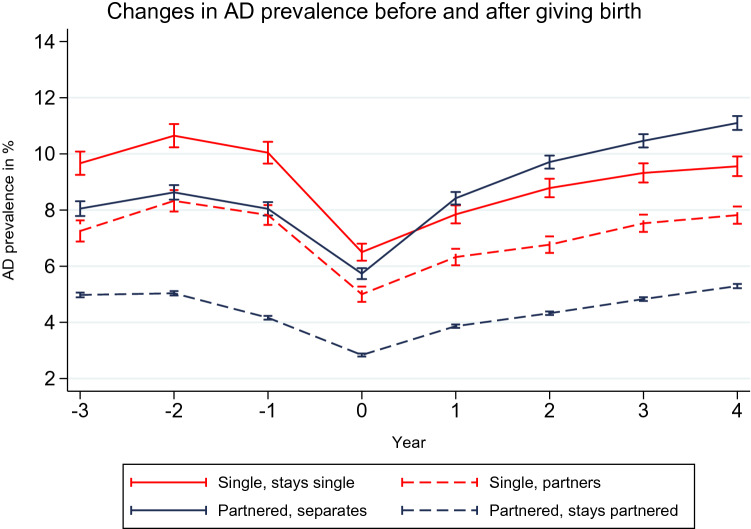
Trajectories of predicted annual antidepressant (AD) prevalence of women giving birth at t_0_, Finland, 1998–2015. *Model is adjusted for age, calendar year, education, individual disposable income, country of birth and municipality group

Figure 3 shows the annual changes in the prevalence of antidepressant use for women who gave birth. The antidepressant trajectories were compared by the women’s partnership status at the time they gave birth and after the birth. Among these women, the prevalence of antidepressant use started declining one year before they gave birth, and reached the lowest level in the year they gave birth. The prevalence of antidepressant purchases among these women clearly increased after they gave birth, and continued to increase during the 5 year follow-up period. This pattern was also reported in a previous study, which found that antidepressant use halved during the first three months of pregnancy, and then increased after pregnancy [53]. The decline in antidepressant use was likely caused by physicians being hesitant to prescribe antidepressants for women who were pregnant or were planning to have children, and by the women’s own fears of the potential effects of antidepressant use on their unborn child.

Our results show that women who entered single motherhood by giving birth and who stayed single had the highest prevalence of antidepressant use in the pre-birth period, with the prevalence reaching a peak of 10.6% (95% CI 10.23–11.06) two years before they gave birth. Even though the prevalence of antidepressant use among these women declined between one year before they gave birth and in the year of the birth, single mothers who stayed single had a prevalence of 6.5% (95% CI 6.19–6.80) in the year they gave birth, which was clearly the highest prevalence among all women who gave birth. Their level of antidepressant use increased again after the birth, and reached a prevalence of 9.6% (95% CI 9.21–9.90) in the fourth post-birth year. Thus, our findings are in line with Hypothesis 3a, which predicted that women who gave birth while single would have worse mental health than their partnered counterparts.

Women who also entered single motherhood through giving birth, but who partnered within the first 5 years after giving birth, had lower levels of antidepressant use than their non-partnered counterparts throughout the post-birth observation period, which is in line with Hypothesis 3b. The prevalence of antidepressant use among these women reached a peak of 8.3% (95% CI 7.95–8.71) two years before the birth, and was 5.0% (95% CI 4.73–5.27) in the year of the birth.

Women who were partnered at the time they gave birth, but who separated from their partner within five years of the birth, had significantly lower levels of antidepressant use in the pre-birth period than single mothers who stayed single. However, because the antidepressant use of these women increased steeply in the years following the birth, by the second post-birth year, they had higher levels of antidepressant use than all of the other categories of women who gave birth.

## Discussion

In this article, we examined mental health inequalities at the intersection of partnership and motherhood biographies that reflect the increasing heterogeneity in family biographies. Thus, we extended the existing research on the mental health disadvantage for single mothers. We investigated the mental health trajectories of women who entered single motherhood through separation, widowhood, or giving birth while single using a unique dataset that provided longitudinal information on the household composition and individual characteristics of these women, as well as an objective measure of their mental health. We documented how experiencing different life events could lead to different mental health trajectories by comparing single mothers to women who also experienced separation, widowhood, or childbirth, but who did not enter single motherhood.

Our findings for all separated women clearly show that their mental health declined before they separated, and remained low in the post-separation period. These results are in line with previous evidence indicating that a separation is a stressful event that is associated with chronic strain both before and after the event [15, 54, 55]. The decline in mental health before the separation may be explained by the anticipation effect that may have resulted from conflicts and stressful interactions with the partner [56]. While we could only observe the years immediately following the separation, our results suggest that the women’s mental health plateaued at a lower overall level, which may indicate that experiencing a separation has longer term effects over the course of a woman’s life.

Although we confirmed that the general pattern was similar for all separated women, we also detected significant differences in the mental health trajectories of different groups of women. We found that women who transitioned to single motherhood had the steepest decline in mental health and significantly lower mental health levels in the initial post-separation years than women who had no underage children. This finding suggests that single mothers may be exposed to cumulative and longer term strains associated with being a single mother that exceed the adverse effects of the separation itself. Our descriptive findings did not indicate that there were considerable differences in the socioeconomic positions of the separated women (see Supplementary Table S1). Thus, in the Finnish context, financial difficulties may be a less important source of strain for single mothers, whereas managing childrearing and work responsibilities, as well as ongoing conflicts with the ex-partner, might be more challenging for single mothers, particularly in the years immediately after the separation.

In addition, this study identified mothers who were not living with their children after separation as a new vulnerable group, as they were the separated women who had the lowest levels of mental health throughout the observation window. The results show that their mental health was already significantly lower than that of all other women who separated as early as three years before the separation, which suggests that these mothers could be a negatively selected group. It is possible that these women gave up or lost custody of their children due to mental health issues or related problems, such as substance abuse. This result is in line with the findings of a study that analyzed the mortality risk of men in Sweden, and found that non-custodial fathers had the greatest mortality risk due to health-related selection [57].

Furthermore, we investigated the effects of re-partnering to explore the dynamic nature of relationships after separation. In addition, taking re-partnering into account gave us information about the duration of single motherhood (more than five years versus less than five years), and thus about the length of the women’s exposure to stressors, which is a key principle of the life course perspective [58]. Our results show that the mental health trajectories of women who re-partnered were better in the post-separation period. This observation is in line with previous evidence indicating that people who divorced and remarried experienced fewer depressive symptoms and less distress than their counterparts who divorced but did not remarry [13, 54]. We advanced these findings by showing that the mental health gap between these groups already existed in the pre-separation period. Therefore, we conclude that our observation that re-partnered women had better mental health than women who remained single can be partially attributed to selection into re-partnering.

Our results for widowed women showed that their antidepressant use increased sharply in the year of the death of their spouse. This observation is in line with the findings of other studies on this topic, which have consistently shown that spousal bereavement is distressing, and has a negative impact on health [21, 22]. The increase in the women’s antidepressant use in the pre-widowhood period might be explained by their anticipation of the loss of their spouse, and by having to cope with a longer period of spousal illness [59].

The only sub-group of widows who had an increased level of antidepressant use in the year following the spousal loss were widowed single mothers who stayed single. This result indicates that the widows in this group suffered from stressors related to both the loss of their partner and having to raise their children on their own, and underlines the importance of considering timing-linked life course contexts [58]. At the same time, widowed single mothers had higher mental health levels than widows without underage biological children. Although the existing studies on childlessness and depression have provided mixed evidence [60], this result indicates that childless women had a mental health disadvantage that narrowed in the post-widowhood period. Thus, the results suggest that widowed single mothers suffered from associated strains over the longer term as well.

The results for re-partnering among widows were mixed. We found that re-partnered single mothers and re-partnered childless women had lower mental health levels in the year they became widowed than those who did not re-partner, which contradicted our expectation that women who re-partnered would have better mental health. However, the age distribution of these women (see Supplementary Table S1) indicated that the re-partnered women were younger than those who did not re-partner. This observation is in line with the previous literature showing that widows who re-partner tend to be younger, and that due to age norms, widowhood [61] is more detrimental at younger than at older ages [22, 62], which underscores the importance of considering the timing of life events. In the post-widowhood period, this gap narrowed and became non-significant. Overall, rather than showing that there was a selection of healthier widows into re-partnering, our results indicate that re-partnering had a positive effect on the mental health of widows.

We also assessed the mental health of an understudied group: namely, women who were single when they gave birth. While the socio-demographic profile of these women differed substantially only from that of women who were partnered when they gave birth and stayed partnered (see Supplementary Table S1*)*, our results suggest that these women were particularly disadvantaged with regard to mental health. The significantly higher use of antidepressants in the year of birth among these women might be explained by their greater exposure to stress. We could not directly test possible explanatory mechanisms using these data. However, in a qualitative study, single mothers reported experiencing higher levels of parenting stress and more distress in the parenting role than married new mothers, which they attributed to receiving less support [63]. Indeed, our results indicated that re-partnered single mothers had significantly lower antidepressant use levels than stably single mothers, which could be attributed to the re-partnered mothers receiving more support. However, the differences in the mental health levels of these two groups were striking even in the pre-birth period, which indicates that there were negative selection processes for those who stayed single and for those who separated.

## Methodological considerations

One of the major strengths of our study is that the large register-based data we used provided information on three important life events—i.e., separation, widowhood, and childbirth—as well as on antidepressant use. The longitudinal study design allowed us to analyze women’s mental health trajectories around these life events, and to estimate the relatively short-term changes in their antidepressant use with minimal loss to follow-up and missing information. Sensitivity analyses that focused on mothers, and were adjusted for receiving a parental allowance or a home care allowance, the number of underage biological children, and the age of the youngest biological child demonstrated the robustness of these patterns (see the supplementary materials, Figure S1–S2). An advantage of our mental health measure was that it was not subject to self-report bias, as the information on antidepressant use was based on registered purchases.

However, this study has some limitations. Registry data do not provide information about different forms of custodial arrangements. Therefore, we could not differentiate between single mothers who were sharing custody with their ex-partner, and those who were not receiving any support from the child’s father. Furthermore, we had no information about the single mothers’ social networks or social support, which might have exerted important protective effects on their well-being. Although we controlled for important characteristics such as socioeconomic status, we had no information about the reasons why the single mothers separated or gave birth. Exiting an unsatisfying relationship might have been less harmful, and thus may have had different effects on single mothers’ mental health that we could not investigate.

Our measure of mental health was based on clinical evaluations. While the measure was objective, it only captured treated individuals. Many people with depressive symptoms do not use antidepressants [46, 47], although in Finland, general practitioners in primary care commonly offer antidepressants to patients seeking help for depressive symptoms [48]. Antidepressants may also be used for non-psychiatric indications, albeit to a lesser extent at working ages [47].

The register-based definition of non-marital cohabiting unions may exclude individuals who are living together but who officially have separate addresses, and it also excludes same-sex couples and couples with an age difference of more than 15 years. These limitations affected our approach to identifying separation and re-partnering events, which was based on the observation that one of the partners moved into or out of the household. Nevertheless, given that the register-based definition resulted in a prevalence of non-marital cohabitating unions that was quite similar to the prevalence obtained in survey samples in Finland, we think that any bias that occurred due to this operationalization of cohabiting was small [16, 64].

## Conclusions

Our results indicated that separation and widowhood had detrimental effects on the mental health of the women in our study sample. These effects were found to be particularly long-lasting for women who entered single motherhood through separation or widowhood, which demonstrates the additional stress and hardship associated with bringing up a child alone. Women who were single at the time of the birth of their child were also identified as a vulnerable group who had relatively high pre-pregnancy as well as post-birth levels of antidepressant use. Re-partnering was associated with better mental health, which could be partially attributed to selection for separated women and for women who gave birth.

Given the growing importance of single-parent households and the increasing heterogeneity among single mothers, these findings emphasize the need to consider different pathways into single motherhood when planning interventions to support the mental health of single mothers, as the levels of social support they already receive and their demands for support might differ. For example, single parenting of very young children is likely to be associated with substantial time constraints and care demands. Health care professionals could help to assess single mothers’ needs, and provide personalized information on support and parenting programs. Widowed single mothers might benefit from bereavement support for both themselves and their grieving children. Ultimately, improving the health of single mothers is also important for the development of their children, as having a mother with poor mental health can have long-lasting adverse consequences for the children’s health. Further research is needed to investigate these associations in contexts where the society offers less institutional support, and where being a single mother is more stigmatized than it is in the Nordic countries. Qualitative studies and research based on survey data could help to further our understanding of whether, and, if so, how social support, perceived financial difficulties, custodial arrangements, and becoming a single mother by choice shapes women’s mental health trajectories.

## Supplementary Information

Below is the link to the electronic supplementary material.

Supplementary file1 (DOCX 168 KB)

## Footnotes

1 Of the women in the sample, 25.5% of the separated women, 0.6% of the widowed women, and 19.9% of the women who gave birth while single experienced more than one life event during the observation window (see Supplementary Table S.2).

## References

[1] Rousou E, Kouta C, Middleton N, Karanikola M (2019). Mental health among single mothers in cyprus: a cross-sectional descriptive correlational study. BMC Womens Health.

[2] Cooper C, Bebbington PE, Meltzer H, Bhugra D, Brugha T, Jenkins R, Farrell M, King M (2008). Depression and common mental disorders in lone parents: results of the 2000 national psychiatric morbidity survey. Psychol Med.

[3] Crosier T, Butterworth P, Rodgers B (2007). Mental health problems among single and partnered mothers. Soc Psychiatry Psychiatr Epidemiol.

[4] Lipman EL, Offord DR, Boyle MH (1997). Single mothers in Ontario: sociodemographic, physical and mental health characteristics. CMAJ.

[5] Wang JL (2004). The difference between single and married mothers in the 12-month prevalence of major depressive syndrome, associated factors and mental health service utilization. Soc Psychiatry Psychiatr Epidemiol.

[6] Kong KA, Choi HY, Kim SI (2017). Mental health among single and partnered parents in South Korea. PLoS ONE.

[7] Collings S, Jenkin G, Carter K, Signal L (2014). Gender differences in the mental health of single parents: New Zealand evidence from a household panel survey. Soc Psychiatry Psychiatr Epidemiol.

[8] Franz M, Lensche H, Schmitz N (2003). Psychological distress and socioeconomic status in single mothers and their children in a German city. Soc Psychiatry Psychiatr Epidemiol.

[9] Afifi TO, Cox BJ, Enns MW (2006). Mental health profiles among married, never-married, and separated/divorced mothers in a nationally representative sample. Soc Psychiatry Psychiatr Epidemiol.

[10] Fritzell S, Gähler M, Fransson E (2020). Child living arrangements following separation and mental health of parents in Sweden. SSM-Population Health.

[11] Statistics Finland (2020) Official Statistics of Finland (OSF): Families [e-publication]. ARAf, Editor http://www.stat.fi/til/perh/2018/02/perh_2018_02_2020-01-31_kat_005_en.html

[12] Härkönen J, Lappalainen E, Jalovaara M (2016). Double disadvantage in a Nordic welfare state: a demographic analysis of the single mother employment gap in Finland, 1987–2011. Stockholm Res Rep Demogr.

[13] Blekesaune M (2008). Partnership transitions and mental distress: investigating temporal order. J Marriage Fam.

[14] Meadows SO, McLanahan SS, Brooks-Gunn J (2008). Stability and change in family structure and maternal health trajectories. Am Sociol Rev.

[15] Metsä-Simola N, Martikainen P (2013). Divorce and changes in the prevalence of psychotropic medication use: a register-based longitudinal study among middle-aged Finns. Soc Sci Med.

[16] Metsä-Simola N, Martikainen P (2014). The effects of marriage and separation on the psychotropic medication use of non-married cohabiters: a register-based longitudinal study among adult Finns. Soc Sci Med.

[17] Strohschein L, McDonough P, Monette G, Shao Q (2005). Marital transitions and mental health: are there gender differences in the short-term effects of marital status change?. Soc Sci Med.

[18] Dziak E, Janzen BL, Muhajarine N (2010). Inequalities in the psychological well-being of employed, single and partnered mothers: the role of psychosocial work quality and work-family conflict. Int J Equity Health.

[19] Avison WR, Ali J, Walters D (2007). Family structure, stress, and psychological distress: a demonstration of the impact of differential exposure. J Health Soc Behav.

[20] Caputo J, Li P, Kühn M, Brønnum-Hansen H, Oksuzyan A (2021). Immigration background and the widowhood effect on mortality. J Gerontol.

[21] Moon JR, Kondo N, Glymour MM, Subramanian SV (2011). Widowhood and mortality: a meta-analysis. PLoS ONE.

[22] Shor E, Roelfs DJ, Curreli M, Clemow L, Burg MM, Schwartz JE (2012). Widowhood and mortality: a meta-analysis and meta-regression. Demography.

[23] Elwert F, Christakis NA (2006). Widowhood and race. Am Sociol Rev.

[24] Carr D, Bodnar-Deren S, Uhlenberg P (2009). Gender, aging and widowhood. International handbook of population aging. International handbooks of population.

[25] Holm AL, Berland AK, Severinsson E (2019). Factors that influence the health of older widows and widowers—a systematic review of quantitative research. Nurs Open.

[26] Woolhouse H, Brown S, Krastev A, Perlen S, Gunn J (2009). Seeking help for anxiety and depression after childbirth: results of the maternal health study. Arch Womens Ment Health.

[27] Howard LM, Molyneaux E, Dennis C-L, Rochat T, Stein A, Milgrom J (2014). Non-psychotic mental disorders in the perinatal period. Lancet.

[28] Gavin NI, Gaynes BN, Lohr KN, Meltzer-Brody S, Gartlehner G, Swinson T (2005). Perinatal depression: a systematic review of prevalence and incidence. Obstet Gynecol.

[29] Agnafors S, Bladh M, Svedin CG, Sydsjö G (2019). Mental health in young mothers, single mothers and their children. BMC Psychiatry.

[30] Saurel-Cubizolles M-J, Romito P, Lelong N, Ancel P-Y (2000). Women’s health after childbirth: a longitudinal study in France and Italy. BJOG: Int J Obstetr Gynaecol.

[31] Don BP, Mickelson KD (2012). Paternal postpartum depression: the role of maternal postpartum depression, spousal support, and relationship satisfaction. Couple Family Psychol.

[32] Elder GH (1994). Time, human agency, and social change: perspectives on the life course. Soc Psychol Quarter.

[33] Elder GH, Johnson MK, Crosnoe R, Shanahan MJ, Mortimer JT (2003). The emergence and development of life course theory. Handbook of the life course.

[34] Elder GH, Kirkpatrick Johnson M (2018) The life course and aging: Challenges, lessons, and new directions. In: Invitation to the life course: Toward new understandings of later life. Routledge, pp 49–81

[35] Glei DA, Goldman N, Weinstein M (2018). Perception has its own reality: subjective versus objective measures of economic distress. Popul Dev Rev.

[36] Mossakowski KN (2008). Is the duration of poverty and unemployment a risk factor for heavy drinking?. Soc Sci Med.

[37] Carr D, Springer KW (2010). Advances in families and health research in the 21st century. J Marriage Fam.

[38] Pevalin DJ, Ermisch J (2004). Cohabiting unions, repartnering and mental health. Psychol Med.

[39] Recksiedler C, Bernardi L (2019). Lone mothers’ repartnering trajectories and health: does the welfare context matter?. J Fam Issues.

[40] Kalmijn M, Monden C (2010). Poverty and union formation among never-married single mothers in the Netherlands, 1989–2005. Popul Stud.

[41] Harkness S, Mortelmans D, Bernardi L (2018). The economic consequences of becoming a lone mother. Lone parenthood in the life course.

[42] Pollmann-Schult M (2018). Single motherhood and life satisfaction in comparative perspective: do institutional and cultural contexts explain the life satisfaction penalty for single mothers?. J Fam Issues.

[43] Cooper CE, McLanahan SS, Meadows SO, Brooks-Gunn J (2009). Family structure transitions and maternal parenting stress. J Marriage Fam.

[44] Ivanova K, Kalmijn M, Uunk W (2013). The effect of children on men's and women's chances of re-partnering in a European context/L'impact des enfants sur les chances d'une nouvelle union pour les hommes et pour les femmes dans un contexte européen. Eur J Population/Revue Européenne de Démographie.

[45] Perelli-Harris B, Amos M (2015). Changes in partnership patterns across the life course: an examination of 14 countries in Europe and the United States. Demogr Res.

[46] Laukkala T, Isometsä E, Hämäläinen J, Heikkinen M, Lindeman S, Aro H (2001). Antidepressant treatment of depression in the Finnish general population. Am J Psychiatry.

[47] Sihvo S, Isometsä E, Kiviruusu O, Hämäläinen J, Suvisaari J, Perälä J, Pirkola S, Saarni S, Lönnqvist J (2008). Antidepressant utilisation patterns and determinants of short-term and non-psychiatric use in the Finnish general adult population. J Affect Disord.

[48] Vuorilehto MS, Melartin TK, Riihimäki K, Isometsä ET (2016). Pharmacological and psychosocial treatment of depression in primary care: low intensity and poor adherence and continuity. J Affect Disord.

[49] Hämäläinen J, Isometsä E, Sihvo S, Kiviruusu O, Pirkola S, Lönnqvist J (2009). Treatment of major depressive disorder in the Finnish general population. Depress Anxiety.

[50] Moustgaard H, Joutsenniemi K, Sihvo S, Martikainen P (2013). Alcohol-related deaths and social factors in depression mortality: a register-based follow-up of depressed in-patients and antidepressant users in Finland. J Affect Disord.

[51] Laaksonen M, Metsä-Simola N, Martikainen P, Pietiläinen O, Rahkonen O, Gould R, Partonen T, Lahelma E (2012). Trajectories of mental health before and after old-age and disability retirement: a register-based study on purchases of psychotropic drugs. Scand J Work Environ Health.

[52] Twisk JWR (2013). Applied longitudinal data analysis for epidemiology: a practical guide.

[53] Jimenez-Solem E, Andersen JT, Petersen M, Broedbaek K, Andersen NL, Torp-Pedersen C, Poulsen HE (2013). Prevalence of antidepressant use during pregnancy in denmark, a nation-wide cohort study. PLoS ONE.

[54] Johnson DR, Wu J (2002). An empirical test of crisis, social selection, and role explanations of the relationship between marital disruption and psychological distress: a pooled time-series analysis of Four-Wave panel data. J Marriage Fam.

[55] Waite L, Luo Y, Lewin A (2009). Marital happiness and marital stability: Consequences for psychological well-being. Soc Sci Res.

[56] Kühn M (2018). Changes in Lone Mothers’ Health: a Longitudinal Analysis. Lone Parenthood in the Life Course.

[57] Ringbäck Weitoft G, Burström B, Rosén M (2004). Premature mortality among lone fathers and childless men. Soc Sci Med.

[58] George LK, Gu D, Dupre ME (2019). George LK (2019) The Life Course Perspective. Encyclopedia of gerontology and population aging.

[59] Carr D, House JS, Wortman C, Nesse R, Kessler RC (2001). Psychological adjustment to sudden and anticipated spousal loss among older widowed persons. J Gerontol.

[60] Quashie NT, Arpino B, Antczak R, Mair CA (2019). Childlessness and health among older adults: variation across five outcomes and 20 countries. J Gerontol.

[61] Martin LG, Binstock RH, George LK (2011). Chapter 3 - demography and aging. Handbook of aging and the social sciences.

[62] Martikainen P, Valkonen T (1996). Mortality after death of spouse in relation to duration of bereavement in Finland. J Epidemiol Community Health.

[63] Copeland D, Harbaugh BL (2005). Differences in parenting stress between married and single first time mothers at six to eight weeks after birth. Issues Compr Pediatr Nurs.

[64] Statistics Finland (2011) Families, 2011. Quality Description, Families, Quality Description F, Editor http://www.stat.fi/til/perh/index_en.html. Accessed Jan 4, 2018

