# Supplementary material for: Pathways into single motherhood, re-partnering, and trajectories of antidepressant medication purchases
Source: Soc Psychiatry Psychiatr Epidemiol. 2022 Oct 13;58(3):409–20. doi: 10.1007/s00127-022-02371-2 (PMC9971110; doi:10.1007/s00127-022-02371-2)
Supplement: Supplementary file 1 — Supplementary file1 (DOCX 168 KB) [file 127_2022_2371_MOESM1_ESM.docx]

Supplementary materials

**Table S1:** Descriptive characteristics

|  | **Separated** | | | | | | **Widowhood** | | | | **Giving birth** | | | |
| --- | --- | --- | --- | --- | --- | --- | --- | --- | --- | --- | --- | --- | --- | --- |
|  | Underage child(ren) | | | | No underage child(ren) | | Underage child(ren) | | No underage child(ren) | | Single | | Partnered | |
|  | Lives with children | | Lives without children | |  |  |  |  |  |  |  |  |  |  |
|  | Single | Re-partnered | Single | Re-partnered | Single | Re-partnered | Single | Re-partnered | Single | Re-partnered | Single | Partnered | Partnered | Single |
| **Mean age, years** | 38.0 | 33.8 | 40.2 | 36.9 | 39.8 | 28.9 | 44.3 | 38.3 | 55.6 | 47.4 | 29.2 | 27.0 | 29.8 | 27.3 |
| **Education** |  |  |  |  |  |  |  |  |  |  |  |  |  |  |
| Low | 20.4 | 24.0 | 23.6 | 23.5 | 22.4 | 15.7 | 19.1 | 21.5 | 34.7 | 32.3 | 33.8 | 33.1 | 9.3 | 25.4 |
| Medium | 43.0 | 45.4 | 39.8 | 40.4 | 49.5 | 57.8 | 43.7 | 46.3 | 42.0 | 45.3 | 41.8 | 43.5 | 39.6 | 48.7 |
| High | 36.6 | 30.7 | 36.6 | 36.0 | 28.1 | 26.5 | 37.2 | 32.3 | 23.3 | 22.4 | 24.4 | 23.5 | 51.2 | 25.9 |
| **Income Quintiles** |  |  |  |  |  |  |  |  |  |  |  |  |  |  |
| 1 | 5.9 | 8.5 | 14.5 | 15.6 | 20.0 | 25.5 | 4.3 | 4.6 | 9.7 | 12.5 | 14.6 | 21.4 | 15.5 | 26.8 |
| 2 | 17.7 | 21.2 | 20.9 | 20.6 | 27.3 | 31.1 | 13.1 | 14.8 | 24.1 | 23.2 | 32.4 | 34.2 | 22.8 | 30.6 |
| 3 | 20.7 | 22.5 | 18.5 | 20.5 | 20.7 | 21.3 | 16.3 | 17.2 | 20.8 | 23.4 | 26.0 | 23.0 | 26.9 | 22.8 |
| 4 | 25.7 | 24.2 | 21.5 | 21.2 | 16.6 | 12.8 | 25.3 | 26.1 | 21.9 | 20.6 | 15.4 | 12.4 | 19.8 | 12.1 |
| 5 | 29.9 | 23.7 | 24.5 | 22.1 | 15.4 | 9.4 | 41.0 | 37.3 | 23.5 | 20.3 | 11.6 | 9.1 | 14.9 | 7.7 |
| **Employment status** |  |  |  |  |  |  |  |  |  |  |  |  |  |  |
| Employed | 69.7 | 67.1 | 63.7 | 67.5 | 65.9 | 79.9 | 76.2 | 72.8 | 61.0 | 67.1 | 44.0 | 45.0 | 69.4 | 52.0 |
| Non-employed | 30.3 | 32.9 | 36.3 | 32.5 | 34.1 | 29.1 | 23.8 | 27.2 | 39.0 | 32.9 | 56.0 | 55.0 | 30.6 | 48.0 |
| Migrant | 9.3 | 8.4 | 12.0 | 8.1 | 6.6 | 5.9 | 6.8 | 5.8 | 3.3 | 5.0 | 10.7 | 8.4 | 6.5 | 8.5 |
| Urban | 71.6 | 68.9 | 71.7 | 70.1 | 76.4 | 80.6 | 57.8 | 57.3 | 61.2 | 63.0 | 76.2 | 68.8 | 68.8 | 72.9 |
| Semi-Urban | 14.7 | 15.8 | 14.5 | 15.0 | 11.7 | 10.2 | 17.8 | 19.5 | 17.5 | 16.4 | 11.9 | 14.4 | 15.4 | 13.8 |
| Rural | 13.8 | 15.3 | 13.8 | 15.0 | 11.9 | 9.2 | 24.5 | 23.2 | 21.3 | 20.6 | 11.9 | 16.8 | 15.8 | 13.3 |

Table S2: Stably single versus re-partnered, descriptive characteristics

|  | Separated | | Widowed | | Giving birth | |
| --- | --- | --- | --- | --- | --- | --- |
| Number of episodes, mean (sd) | 1.25 (0.57) | | 1.01 (0.08) | | 1.33 (0.6) | |
| Number of episodes = 1, percentage | 74.5 | | 99.4 | | 80.1 | |
|  | stably single | re-partnered | stably single | re-partnered | stably single | re-partnered |
| Age at event, mean (sd) | 39.5 (12.2) | 29.4 (9.26) | 53.3 (7.8) | 44.1 (10.7) | 29.2 (6.9) | 26.5 (6.6) |
| Age at re-partnering, mean (sd) |  | 30.9 (9.24) |  | 45.8 (10.9) |  | 27.8 (6.5) |
| Years from event to re-partnering, mean (sd) |  | 1.7 (1.2) |  | 2.03 (1.2) |  | 1.36 (1.3) |

Table S3: Logistic regression model, separated women

|  | **Odds Ratio** | **95 % Conf. Interval** | |
| --- | --- | --- | --- |
| **Age** | 1.016545 | 1.016034 | 1.017057 |
| **Calendar year** | 1.02881 | 1.027673 | 1.029949 |
| **Education (Ref. Low)** |  |  |  |
| Middle | 0.89421 | 0.884104 | 0.904432 |
| High | 0.83515 | 0.823578 | 0.846884 |
| **Disposable income quintiles (Ref. 1)** |  |  |  |
| 2 | 1.173611 | 1.163616 | 1.183692 |
| 3 | 1.216936 | 1.204671 | 1.229326 |
| 4 | 1.211589 | 1.197861 | 1.225474 |
| 5 | 1.215634 | 1.200141 | 1.231326 |
| **Employment status (Ref. Not employed)** |  |  |  |
| Employed | 0.759128 | 0.753221 | 0.76508 |
| **Unemployment (Ref. Not unemployed)** |  |  |  |
| Unemployed | 0.919243 | 0.910605 | 0.927963 |
| **Country of birth (Ref. Finland)** |  |  |  |
| Not Finland | 0.561209 | 0.546229 | 0.5766 |
| **Municipality groups (Ref. Urban)** |  |  |  |
| Semi-Urban | 0.947969 | 0.938036 | 0.958007 |
| Rural | 0.946533 | 0.936231 | 0.956949 |
| **Life event groups (Ref. No underage biological child(ren), stays single)** |  |  |  |
| No underage biological child(ren), re-partners | 0.693458 | 0.676142 | 0.711218 |
| Lives with children, stays single | 0.869096 | 0.845279 | 0.893583 |
| Lives with children, re-partners | 0.780779 | 0.756625 | 0.805704 |
| Not with children, stays single | 1.482536 | 1.415729 | 1.552496 |
| Not with children, re-partners | 1.142645 | 1.090193 | 1.197621 |
| **Years around the life-event (Ref. -3)** |  |  |  |
| -2 | 1.064516 | 1.047052 | 1.08227 |
| -1 | 1.168873 | 1.149959 | 1.188098 |
| 0 | 1.427448 | 1.404836 | 1.450505 |
| 1 | 1.330493 | 1.30887 | 1.352474 |
| 2 | 1.22876 | 1.208074 | 1.2498 |
| 3 | 1.182747 | 1.161909 | 1.203958 |
| 4 | 1.15574 | 1.134471 | 1.177407 |
| **Life event groups#Years around the life-event** |  |  |  |
| No underage biological child(ren), re-partners#-2 | 1.063364 | 1.037215 | 1.090172 |
| No underage biological child(ren), re-partners#-1 | 1.125527 | 1.098584 | 1.153131 |
| No underage biological child(ren), re-partners#0 | 1.085822 | 1.060614 | 1.111629 |
| No underage biological child(ren), re-partners#1 | 1.106296 | 1.080505 | 1.132702 |
| No underage biological child(ren), re-partners#2 | 1.194671 | 1.166617 | 1.223399 |
| No underage biological child(ren), re-partners#3 | 1.231331 | 1.201889 | 1.261493 |
| No underage biological child(ren), re-partners#4 | 1.288767 | 1.257444 | 1.32087 |
| Lives with children, stays single#-2 | 1.037314 | 1.009066 | 1.066352 |
| Lives with children, stays single#-3 | 1.109294 | 1.079969 | 1.139416 |
| Lives with children, stays single#0 | 1.226586 | 1.195413 | 1.258572 |
| Lives with children, stays single#1 | 1.207856 | 1.177048 | 1.239471 |
| Lives with children, stays single#2 | 1.192056 | 1.161366 | 1.223556 |
| Lives with children, stays single#3 | 1.196795 | 1.165405 | 1.229031 |
| Lives with children, stays single#4 | 1.200127 | 1.168141 | 1.232989 |
| Lives with children, re-partners#-2 | 1.076998 | 1.044121 | 1.110912 |
| Lives with children, re-partners-1 | 1.155042 | 1.120857 | 1.19027 |
| Lives with children, re-partners#0 | 1.212032 | 1.177464 | 1.247616 |
| Lives with children, re-partners#1 | 1.181145 | 1.147327 | 1.21596 |
| Lives with children, re-partners#2 | 1.205539 | 1.170779 | 1.24133 |
| Lives with children, re-partners#3 | 1.273501 | 1.236405 | 1.31171 |
| Lives with children, re-partners#4 | 1.290148 | 1.252127 | 1.329325 |
| Not with children, stays single#-2 | 1.016149 | 0.970246 | 1.064224 |
| Not with children, stays single#-1 | 1.097774 | 1.04957 | 1.148191 |
| Not with children, stays single#0 | 1.034601 | 0.990209 | 1.080983 |
| Not with children, stays single#1 | 0.978786 | 0.936191 | 1.023319 |
| Not with children, stays single#2 | 0.958786 | 0.916561 | 1.002955 |
| Not with children, stays single#3 | 0.941234 | 0.898839 | 0.98563 |
| Not with children, stays single#4 | 0.931898 | 0.889057 | 0.976802 |
| Not with children, re-partners#-2 | 1.080445 | 1.031242 | 1.131996 |
| Not with children, re-partners#-1 | 1.154779 | 1.103705 | 1.208217 |
| Not with children, re-partners#0 | 1.095632 | 1.048385 | 1.145008 |
| Not with children, re-partners#1 | 1.010836 | 0.966566 | 1.057133 |
| Not with children, re-partners#2 | 1.021108 | 0.976052 | 1.068245 |
| Not with children, re-partners#3 | 1.035641 | 0.989513 | 1.083918 |
| Not with children, re-partners#4 | 1.067626 | 1.019705 | 1.117798 |
| **Cons** | 1.43E-26 | 1.56E-27 | 1.31E-25 |
| **Number of observations** | 4,548,025 |  |  |
| **Number of groups** | 627,586 |  |  |
|  |  |  |  |
|  |  |  |  |
|  |  |  |  |
|  |  |  |  |

Table S4: Logistic regression model, widowed women

|  | **Odds Ratio** | **95 % Conf. Interval** | |
| --- | --- | --- | --- |
| **Age** | 0.988247 | 0.985247 | 0.991256 |
| **Calendar year** | 1.021807 | 1.017145 | 1.026491 |
| **Education (Ref. Low)** |  |  |  |
| Middle | 1.032771 | 0.985271 | 1.082562 |
| High | 1.026878 | 0.970669 | 1.086342 |
| **Disposable income quintiles (Ref. 1)** |  |  |  |
| 2 | 1.04317 | 1.007315 | 1.080302 |
| 3 | 1.031463 | 0.990749 | 1.073851 |
| 4 | 1.026761 | 0.983074 | 1.07239 |
| 5 | 1.039518 | 0.992157 | 1.08914 |
| **Employment status (Ref. Not employed)** |  |  |  |
| Employed | 0.746159 | 0.725124 | 0.767804 |
| **Unemployment (Ref. Not unemployed)** |  |  |  |
| Unemployed | 0.835231 | 0.808541 | 0.862803 |
| **Country of birth (Ref. Finland)** |  |  |  |
| Not Finland | 0.622074 | 0.551506 | 0.701672 |
| **Municipality groups (Ref. Urban)** |  |  |  |
| Semi-Urban | 0.942728 | 0.904051 | 0.98306 |
| Rural | 0.910729 | 0.873811 | 0.949207 |
| **Life event groups (Ref. No underage biological child(ren), stays single** |  |  |  |
| No underage biological child(ren), re-partners | 0.978622 | 0.877008 | 1.09201 |
| Lives with children, stays single | 0.605506 | 0.546089 | 0.671388 |
| Lives with children, re-partners | 0.612963 | 0.51395 | 0.73105 |
| **Years around the life-event (Ref. -3)** |  |  |  |
| -2 | 1.067441 | 1.036818 | 1.098969 |
| -1 | 1.148123 | 1.110072 | 1.187479 |
| 0 | 1.786435 | 1.719183 | 1.856319 |
| 1 | 1.688099 | 1.619534 | 1.759567 |
| 2 | 1.395359 | 1.334087 | 1.459445 |
| 3 | 1.236249 | 1.176792 | 1.29871 |
| 4 | 1.177434 | 1.116138 | 1.242097 |
| **Life event groups#Years around the life-event** |  |  |  |
| No underage biological child(ren), re-partners#-2 | 1.060706 | 0.976784 | 1.151838 |
| No underage biological child(ren), re-partners#-1 | 1.152098 | 1.050741 | 1.263233 |
| No underage biological child(ren), re-partners#0 | 1.242217 | 1.123018 | 1.374067 |
| No underage biological child(ren), re-partners#1 | 1.145172 | 1.032193 | 1.270518 |
| No underage biological child(ren), re-partners#2 | 1.035539 | 0.929842 | 1.153251 |
| No underage biological child(ren), re-partners#3 | 1.061953 | 0.949124 | 1.188194 |
| No underage biological child(ren), re-partners#4 | 1.108056 | 0.986977 | 1.243989 |
| Lives with children, stayes single#-2 | 1.063796 | 0.988752 | 1.144536 |
| Lives with children, stayes single#-1 | 1.116349 | 1.028278 | 1.211963 |
| Lives with children, stayes single#0 | 1.413977 | 1.292772 | 1.546546 |
| Lives with children, stayes single#1 | 1.560148 | 1.423942 | 1.709382 |
| Lives with children, stayes single#2 | 1.528761 | 1.39212 | 1.678814 |
| Lives with children, stayes single#3 | 1.532501 | 1.389779 | 1.689879 |
| Lives with children, stayes single#4 | 1.546147 | 1.397648 | 1.710423 |
| Lives with children, re-partners#-2 | 1.153803 | 1.017971 | 1.307759 |
| Lives with children, re-partners#-1 | 1.267002 | 1.101472 | 1.457408 |
| Lives with children, re-partners#0 | 1.482822 | 1.270622 | 1.730461 |
| Lives with children, re-partners#1 | 1.414195 | 1.206967 | 1.657004 |
| Lives with children, re-partners#2 | 1.270098 | 1.078569 | 1.495638 |
| Lives with children, re-partners#3 | 1.445358 | 1.222197 | 1.709265 |
| Lives with children, re-partners#4 | 1.270326 | 1.066658 | 1.512884 |
| **Cons** | 4.78E-20 | 5.21E-24 | 4.38E-16 |
| **Number of observations** | 305,403 |  |  |
| **Number of groups** | 43,165 |  |  |

Table S5: Logistic regression model, birth giving women

|  | **Odds Ratio** | **95 % Conf. Interval** | |
| --- | --- | --- | --- |
| **Age** | 1.028681 | 1.02711 | 1.030255 |
| **Calendar year** | 1.053865 | 1.052219 | 1.055514 |
| **Education (Ref. Low)** |  |  |  |
| Middle | 0.803887 | 0.788378 | 0.819702 |
| High | 0.631794 | 0.617346 | 0.646581 |
| **Disposable income quintiles (Ref. 1)** |  |  |  |
| 2 | 1.076457 | 1.062492 | 1.090605 |
| 3 | 1.090191 | 1.073969 | 1.106658 |
| 4 | 1.113871 | 1.095221 | 1.132839 |
| 5 | 1.123263 | 1.101511 | 1.145443 |
| **Employment status (Ref. Not employed)** |  |  |  |
| Employed | 0.903504 | 0.892896 | 0.914239 |
| **Unemployment (Ref. Not unemployed)** |  |  |  |
| Unemployed | 1.094882 | 1.078198 | 1.111825 |
| **Country of birth (Ref. Finland)** |  |  |  |
| Not Finland | 0.513873 | 0.49436 | 0.534157 |
| **Municipality groups (Ref. Urban)** |  |  |  |
| Semi-Urban | 0.924855 | 0.909933 | 0.940022 |
| Rural | 0.906619 | 0.890899 | 0.922616 |
| **Life event groups (Ref. Single, stays single)** |  |  |  |
| Single, partners | 0.728492 | 0.67742 | 0.783415 |
| Partnered, stays partnered | 0.486276 | 0.46244 | 0.511341 |
| Partnered, separates | 0.816521 | 0.770216 | 0.865611 |
| **Years around the life-event (Ref. -3)** |  |  |  |
| -2 | 1.115119 | 1.06038 | 1.172685 |
| -1 | 1.04387 | 0.99277 | 1.097599 |
| 0 | 0.646987 | 0.612775 | 0.683109 |
| 1 | 0.793566 | 0.753664 | 0.835581 |
| 2 | 0.89884 | 0.854591 | 0.94538 |
| 3 | 0.960273 | 0.912668 | 1.01036 |
| 4 | 0.987405 | 0.937731 | 1.03971 |
| **Life event groups#Years around the life-event** |  |  |  |
| Single, partners#-2 | 1.043142 | 0.965947 | 1.126506 |
| Single, partners#-1 | 1.040341 | 0.963775 | 1.12299 |
| Single, partners#0 | 1.037223 | 0.955259 | 1.126221 |
| Single, partners#1 | 1.086639 | 1.006076 | 1.173653 |
| Single, partners#2 | 1.031525 | 0.956974 | 1.111883 |
| Single, partners#3 | 1.084424 | 1.006822 | 1.168009 |
| Single, partners#4 | 1.099142 | 1.020468 | 1.183882 |
| Partnered, stays partnered#-2 | 0.908117 | 0.861063 | 0.957743 |
| Partnered, stays partnered#-1 | 0.794313 | 0.753242 | 0.837623 |
| Partnered, stays partnered#0 | 0.859386 | 0.811602 | 0.909984 |
| Partnered, stays partnered#1 | 0.965683 | 0.915239 | 1.018906 |
| Partnered, stays partnered#2 | 0.95955 | 0.910967 | 1.010725 |
| Partnered, stays partnered#3 | 1.008714 | 0.957805 | 1.062329 |
| Partnered, stays partnered#4 | 1.081035 | 1.026323 | 1.138663 |
| Partnered, separates#-2 | 0.967925 | 0.909693 | 1.029884 |
| Partnered, separate#-1 | 0.956957 | 0.899638 | 1.017928 |
| Partnered, separate#0 | 1.070275 | 1.001795 | 1.143436 |
| Partnered, separate#1 | 1.323873 | 1.244925 | 1.407827 |
| Partnered, separate#2 | 1.369397 | 1.290156 | 1.453506 |
| Partnered, separate#3 | 1.394636 | 1.314238 | 1.479951 |
| Partnered, separate#4 | 1.450077 | 1.366319 | 1.538969 |
| **Cons** | 1.25E-47 | 5.44E-49 | 2.88E-46 |
| **Number of observations** | 3,992,688 |  |  |
| **Number of groups** | 515,583 |  |  |

Figure S1: Prevalence of antidepressant use of women separating at t


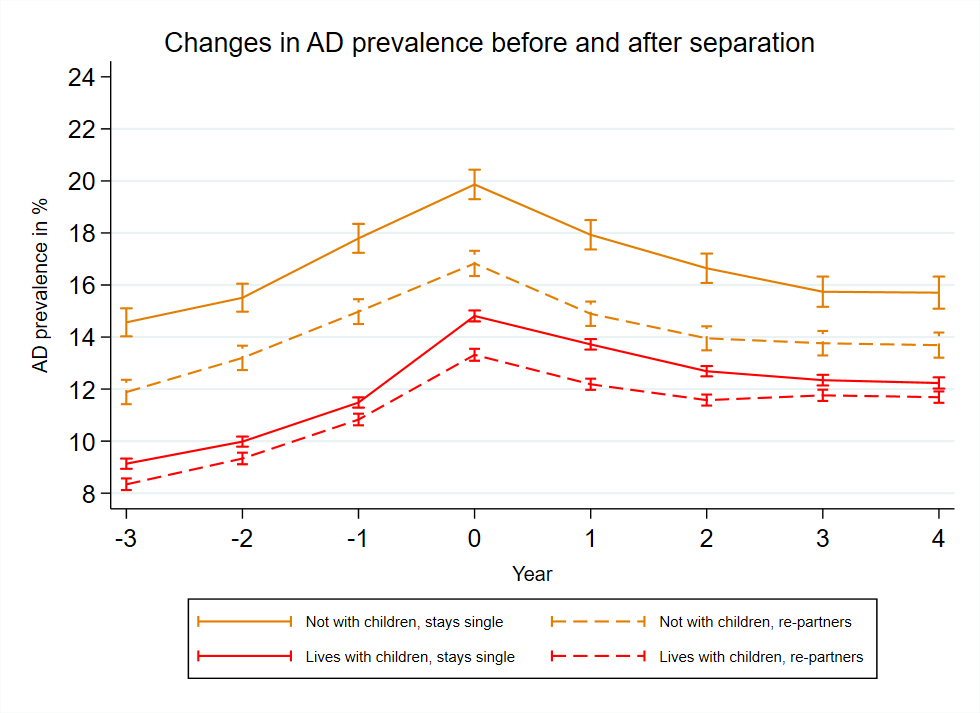


**Adjusted for age, calendar year, SES, migrant status, urban/rural area, parental allowance, home allowance, number of the children and age of youngest biological child.*

**Figure S2:** Prevalence of antidepressant use of women becoming widowed at t_0_


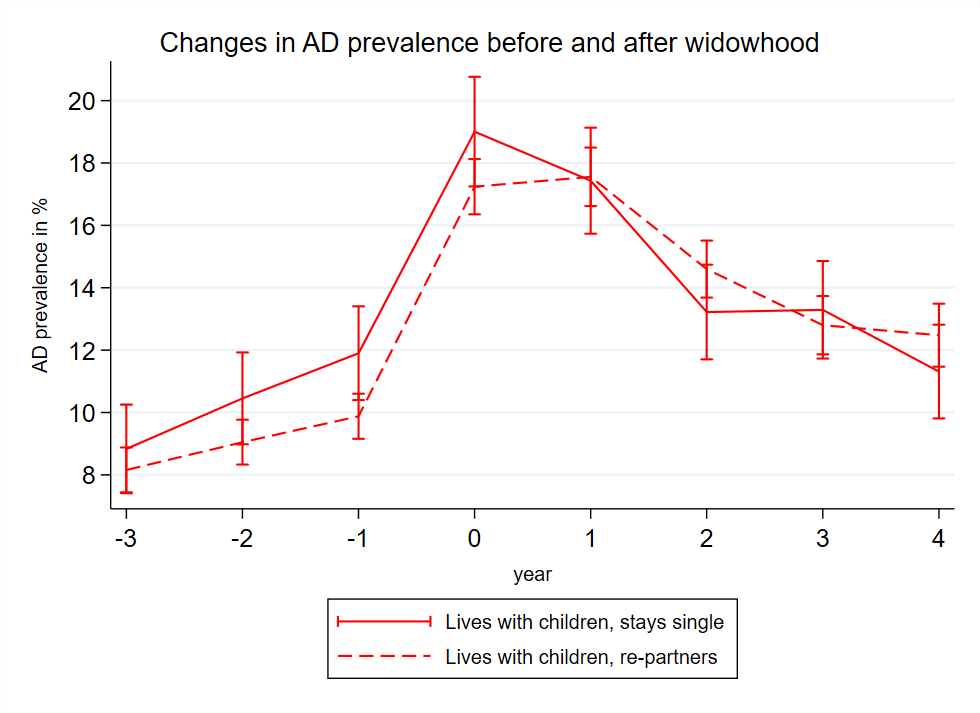


**Adjusted for age, calendar year, SES, migrant status, urban/rural area, parental allowance, home allowance, number of the children and age of youngest biological child.*
